# Translating medical documents improves students’ communication skills in simulated physician-patient encounters

**DOI:** 10.1186/s12909-016-0594-4

**Published:** 2016-02-27

**Authors:** Anja Bittner, Johannes Bittner, Ansgar Jonietz, Christoph Dybowski, Sigrid Harendza

**Affiliations:** “Was hab ich?” gGmbH, Theaterstraße 4, D-01067 Dresden, Germany; Universitätsklinikum Hamburg-Eppendorf, III. Medizinische Klinik, Martinistr. 52, D-20246 Hamburg, Germany

**Keywords:** Communication skills, Objective structured clinical examination (OSCE), Physician-patient encounter, Simulated patients, Undergraduate medical education

## Abstract

**Background:**

Patient-physician communication should be based on plain and simple language. Despite communication skill trainings in undergraduate medical curricula medical students and physicians are often still not aware of using medical jargon when communicating with patients. The aim of this study was to compare linguistic communication skills of undergraduate medical students who voluntarily translate medical documents into plain language with students who do not participate in this voluntary task.

**Methods:**

Fifty-nine undergraduate medical students participated in this study. Twenty-nine participants were actively involved in voluntarily translating medical documents for real patients into plain language on the online-platform https://washabich.de (WHI group) and 30 participants were not (non-WHI group). The assessment resembled a virtual consultation hour, where participants were connected via skype to six simulated patients (SPs). The SPs assessed participants’ communication skills. All conversations were transcribed and assessed for communication skills and medical correctness by a blinded expert. All participants completed a self-assessment questionnaire on their communication skills.

**Results:**

Across all raters, the WHI group was assessed significantly (*p* = .007) better than the non-WHI group regarding the use of plain language. The blinded expert assessed the WHI group significantly (*p* = .018) better regarding the use of stylistic devices of communication. The SPs would choose participants from the WHI group significantly (*p* = .041) more frequently as their personal physician. No significant differences between the two groups were observed with respect to the medical correctness of the consultations.

**Conclusion:**

Written translation of medical documents is associated with significantly more frequent use of plain language in simulated physician-patient encounters. Similar extracurricular exercises might be a useful tool for medical students to enhance their communication skills with respect to using plain language in physician-patient communication.

**Electronic supplementary material:**

The online version of this article (doi:10.1186/s12909-016-0594-4) contains supplementary material, which is available to authorized users.

## Background

Every physician should use plain and simple language while communicating with patients. Providing simplified information leads to a better understanding in patients [1] which is the inevitable basis for shared decision-making [2] and health literacy – the ability to understand and apply medical information [3]. Berkman et. al. found low health literacy to be associated with more hospitalizations, poorer overall health status and even higher mortality rates [4]. Another systematic review showed a significant association between clinical outcomes and physicians’ use of plain language [5].

However, lay people often do not fully comprehend medical information provided due to medical jargon being used by their physicians when talking to them [6]. Furthermore, medical jargon can be perceived as having negative connotations or may be understood in an unintended way by lay people [7]. Nevertheless, physicians tend to overestimate the clarity of their communication when talking with patients [8]. It was shown that physicians use medical jargon frequently [9] and leave it unexplained [10]. For example, residents asked to talk with standardized patients about breast cancer or prostate cancer explained only 15 percent of the medical terms used in these conversations [10].

Meanwhile, many medical schools offer communication skills trainings in their undergraduate curricula to overcome this issue [11–13]. Furthermore, standards and checklists have been developed to improve communication skills in doctor-patient interactions [14]. At one medical school, students were requested to write letters to patients as a teaching tool for communication focussing on the doctor-patient-relationship and on the use of plain language when interacting with patients [15]. With this teaching tool, students’ awareness of medical jargon or technical terms unclear to a lay reader was significantly sharpened [15]. As several studies indicate that peer-teaching [16, 17], longitudinal courses [18] and self-directed learning [17, 19] has a positive impact on the learning results of medical students with respect to communication or practical skills, these concepts might improve medical students’ use of plain language, too.

On the website https://washabich.de [“what’s my diagnosis”] German speaking medical students (year four or higher) can voluntarily translate medical documents for patients into plain language [20]. A pilot study has shown that translating medical documents on this website enhances students’ written communications skills [21]. Besides translating medical documents into plain language, the volunteers also often have to investigate special medical knowledge to translate the medical documents sent in by the patients correctly. If working with this website were demonstrated to have an impact on medical students’ communication skills in direct physician-patient interactions, it would be a valuable tool for undergraduate medical education.

Usually communication skills can be assessed with objective structured clinical examinations (OSCEs) that mostly focus on specific situations in medical encounters like “breaking bad news” using specific checklists [22, 23]. Standardized patients’ views were demonstrated to be an important additional feature for OSCE construction as their ratings include the core process of doctor-patient relationship building, which adds a more realistic dimension to the assessment [24]. However, this underscores the need to develop a tool assessing the usage of plain language in physician-patient encounters as the key component of interaction. As modern physician-patient communication also increasingly includes telemedical settings [25] it might be a useful approach to create an online assessment for communication skills, which serves to examine medical students independently from the location of their medical school.

We hypothesize that translating medical documents into plain language voluntarily for the website https://washabich.de increases the volunteers’ skills for good doctor-patient communication with a special focus on using plain language in oral patient encounters. We also hypothesize, that voluntary translation of medical documents into plain language increases medical students knowledge about diseases and treatment. Furthermore, we hypothesize that patients are overall more satisfied with their encounters with washabich-volunteers. We tested these hypotheses in a pilot project with a new OSCE-like format to assess communication skills.

## Methods

### Online assessment

We developed an online assessment as a quasi-experimental study design with an intervention group and a control group to evaluate undergraduate medical students’ (year four or higher) communication skills with respect to patient centered use of plain language and correct consulting. In a first step, we assembled six medical reports comparable to original reports submitted to the online-platform washabich.de most frequently. These included: 1) X-ray of the lumbar spine, 2) cardiac catheterization, 3) magnet resonance imaging (MRI) of the knee, 4) abdominal ultrasound, 5) histology of a colon polyp, 6) laboratory results of chronic renal disease. Secondly, we designed patient cases for each of these reports and trained six experienced actors from the standardized patient (SP) program of the Medical Faculty of the Dresden University of Technology for their respective role. Additionally, each SP was instructed to ask specific questions if a given information regarding his/her medical report or information on consequences were incomprehensible or incomplete.

The assessment resembled a virtual consultation hour in a general practitioner’s practice. Participating students were connected via Skype from their homes to six computers in Dresden where the SPs rotated after each consultation. The participants’ assignment was as follows: “You are a resident in Dr. Buechner’s practice. It’s a busy day and Dr. Buechner has asked you to call some of his patients via Skype to explain the results of their new medical reports to them and to discuss the consequences and next steps if necessary.” Before each consultation, participants were given five minutes to read the next patient’s report and a brief medical chart online. Afterwards, participants talked to the SPs for ten minutes. All conversations were recorded as MP3-files. After each consultation, every SP completed a questionnaire with items regarding the participant’s communication skills. All SPs had received a frame-of-reference training [26] two weeks prior to the assessment. This training included explanations about the use of scoring forms and the impact of scoring biases to set equal assessment standards.

After the sixth conversation, participants filled in a self-assessment questionnaire regarding their communication and consultation skills. The recorded conversations were transcribed verbatim. We developed a scoring system to assess the consultation and communications skills from the transcripts similar to the SPs’ questionnaires and the self-assessment of the participants. Correctness of the medical information given to the SP was assessed with a score as well. This score was different for the six cases with respect to the medical requirements of each case.

### Questionnaires

Each SP completed one questionnaire for every candidate after every consultation to assess the quality of communication. This questionnaire was newly assembled for the purpose of our study from different questionnaires for communication skills [27–29]. It included nine items, seven of them targeting communication and consultation skills and two items comprising general statements about the overall satisfaction with the counselling and with the participant as a physician. The seven items targeting communication and consultation skills assessed three different categories: use of plain language, use of stylistic devices of communication, and subjective comprehensibility (Table 1). All items were assessed on a 5-point Likert scale (1: I strongly disagree, 2: I disagree, 3: I neither disagree nor agree, 4: I agree, 5: I strongly agree).

**Table 1:** Assessment of communication and consultation skills

	Patient	Expert	Participants
Plain language	The participant used plain language.	The participant used plain language.	I used plain language.
	The participant explained medical terms.	The participant explained medical terms.	I explained the medical terms.
	The participant explained the meaning of the medical report.	The participant explained the meaning of the medical report.	I explained the meaning of the medical report.
Stylistic device of communication	The participant asked me whether I had understood the explanations.	The participant asked the patient whether he had understood the explanations.	I asked the patients whether they had understood the explanations.
	The participant encouraged me to ask questions.	The participant encouraged the patient to ask questions.	I encouraged the patients to ask questions.
Subjective comprehensibility	The participant answered my questions satisfactory.	The participant answered the patients’ questions satisfactory.	I answered the questions satisfactory.
	The participant comprehensibly explained the next steps of diagnostic or therapeutic procedures.	The participant comprehensibly explained the next steps of diagnostic or therapeutic procedures.	I comprehensibly explained the next steps of diagnostic or therapeutic procedures.
General statements	I am satisfied with the medical counselling.		I am satisfied with the medical counseling for my patients.
	I would choose this participant as my personal physician.		

The candidates completed a similar self-assessment questionnaire on their communication and consultation skills after the sixth consultation. This questionnaire included the seven items of the SP-questionnaire targeting the three categories of communication and consultation skills and one additional item inquiring about the overall satisfaction with his/her own consultation skills (Table 1).

Two scores were developed for assessing the transcripts. The first score included the seven items on communication and consultation skills used in the questionnaires of the SPs and the participants (Table 1). The second score targeted the medical correctness of the given information. The respective items were different for the six medical cases and based on information given for each case by a medical specialist from the University Medical Center Hamburg-Eppendorf. This score included five items per case. They were assessed on a 4-point rating scale (0: not explained, 1: correctly explained on demand, 2: incompletely explained, 3: correctly explained). As a separate sixth item, serious medical mistakes were counted and later subtracted from the sum of the other five items. The participants could obtain a maximum of 15 points per case for the medical correctness. A blinded medical expert not involved in the study design and with special training for assessing communication skills performed the scoring of the transcripts in randomized order. All questionnaires can be found in the Additional file 1.

### Participants

Fifty-nine undergraduate medical students from 23 German medical schools participated in the assessment. Participants’ characteristics are shown in Table 2. The intervention group (WHI group) included 29 participants who were actively involved in voluntary work with the online-platform washabich.de during their undergraduate medial education. While working with this platform, these participants had translated 78.7 ± 28.4 medical documents on average into plain language until the time of this study. The control group (non-WHI group) consisted of 30 participants who were not involved in translation work with the online-platform https://washabich.de. Participants of the non-WHI group had been recruited by the participating students from the WHI group. If possible, non-WHI participants were chosen from the same medical school, matching semester and gender. All students were informed that we wished to test a new online OSCE format to simulate physician-patient encounters. They did not receive any information on the individual assessment criteria themselves. One participant of the non-WHI group was excluded from data analysis after the assessment because he did not speak German fluently. All participants were at least in their fourth academic year of medical studies or had just finished medical school but had not started to work as a physician at the time of the assessment. Participants were assigned randomly to a consultation hour. Each consulting hour lasted 90 min. A member of the State of Hamburg Physicians’ Ethics Board reviewed and approved this study. Informed consent was signed by all participants and their anonymity was guaranteed.

**Table 2:** Socio-demographic data

	WHI	Non-WHI
	(*N* = 29)	(*N* = 29)
Age in years (M ± SD)	25.1 ± 3.1	25.2 ± 2.2
Gender		
Female participants (%)	86.2	75.9
Male participants (%)	13.8	24.1
Semester of undergraduate medical training (M ± SD)	11.0 ± 1.7	10.2 ± 1.6
German language ability		
Mother tongue, accent-free or comparable (%)	100	96.6
Fluent with accent (%)	0	3.4

### Data analysis

Statistical analyses were performed using IBM SPSS Statistics 22.0 (Armonk, NY: IBM Corp.). Demographic data of the WHI and the non-WHI group were compared with t-tests, Chi-square tests, and exact Fisher’s exact tests depending on the scale characteristic of a specific item. The use of plain language was defined as the primary outcome. Further beneficial communication skills, medical correctness as well as patient satisfaction with the medical counselling and patient preference for a specific physician were defined as secondary outcomes. To compare the questionnaire and score results of the WHI and the non-WHI group we used analyses of covariance (MANCOVAs and ANCOVAs). Even though the difference for the semesters of undergraduate training was not significant between the two groups (*p* = .07) we used “semester of participant” as a covariate in all group comparisons to exclude any potential bias. The level of significance for all findings was set to *p* < .05. To ascertain the impact of the significant differences we also calculated effect sizes (Cohens’ d, Cramer’s Phi or partial eta-squared) depending on the used statistical test. Furthermore, we calculated Pearson correlation coefficients for the communication skills and “satisfaction with the participants as physicians” assessed in the SPs’ questionnaire.

## Results

The socio-demographic data of the WHI group (intervention group) did not differ significantly from those of the non-WHI group (Table 2). In both groups, the percentage of female participants was high (86.2 % in the WHI group and 75.9 % in the non-WHI group). Across all raters, the WHI group was assessed significantly (*p* = .007) better than the non-WHI group (control group) with respect to the use of plain language, with group affiliation explaining 22.5 % of the unexplained variance (partial η^2^ = .225; Table 3). Additionally, the blinded expert assessed the WHI group significantly (*p* = .018) better than the non-WHI group with respect to the use of stylistic devices of communication, with group affiliation explaining 11.3 % of the unexplained variance (partial η^2^ = .113).

**Table 3:** Comparison of communication and consultation skills of WHI and non-WHI group

	Standardized patient		Expert		Self-assessment		Sum/MANCOVA	
	WHI	Non-WHI	WHI	Non-WHI	WHI	Non-WHI	*p*	Partial η^2^
	(Est. M/SE)	(Est. M/SE)	(Est. M/SE)	(Est. M/SE)	(Est. M/SE)	(Est. M/SE)		
Plain language	4.13*/0.10	3.80/0.10	3.09^#^/0.09	2.69/0.10	4.11*/0.10	3.74/0.10	.007^#^	.225
Stylistic device of communication	3.53/0.11	3.34/0.12	2.46*/0.09	2.12/0.10	3.47/0.17	3.53/0.17	.112	.123
Subjective comprehensibility	4.01/0.11	3.78/0.11	2.82/0.07	2.83/0.08	3.54/0.15	3.14/0.15	.262	.084

*p < 0.05, ^#^ *p* < 0.01

While no significant difference between the groups was found for the SPs’ satisfaction with the medical counselling, the SPs indicated to choose participants from the WHI group significantly (*p* = .041) more frequently as their personal physician (Table 4), with group affiliation explaining 7.4 % of the unexplained variance (partial η^2^ = .074). For this item (“I would choose this participant as my personal physician.”) we found significant correlations (*p* < .001) with the SPs’ ratings in the three different categories of communication skills across both groups (use of plain language, r = .859; use of stylistic devices of communication, r = .593; subjective comprehensibility, r = .888). No significant differences between the two groups were observed for the medical correctness of the consultations (Table 5).

**Table 4:** SPs’ general statements

General statements (SPs)	WHI	Non-WHI
	(Est. M/SE)	(Est. M/SE)
I am satisfied with the medical counselling	3.79/0.13	3.44/0.13
I would choose this participant as my personal physician	3.65*/0.14	3.23/0.14

*p < 0.05

**Table 5:** Medical correctness

Medical correctness	WHI	Non-WHI	*p*
	(Est. M/SE)	(Est. M/SE)	
Case 1: X-ray of the lumbar spine	6.46/0.42	6.83/0.42	.54
Case 2: Cardiac catheter examination	11.21/0.57	11.07/0.57	.86
Case 3: MRI of the knee	6.79/0.59	6.91/0.60	.88
Case 4: Abdominal ultrasound	4.20/0.87	5.01/0.90	.52
Case 5: Histology of a colon polyp	7.28/0.62	5.89/0.62	.12
Case 6: Laboratory results of chronic renal disease	1.53/0.65	3.28/0.67	.07

## Discussion

The findings of this study suggest that voluntary translation of written medical documents into plain language for real patients is associated with a significantly greater use of plain language in simulated oral physician-patient encounters. This is an intriguing finding because other studies show that the transfer of skills or knowledge between contexts is difficult for learners [30, 31]. In contrast to other studies and to mandatory communication courses, WHI students participate voluntarily in the translating service for patients on the website https://washabich.de. Writing letters to patients has been shown to be a useful tool for medical students to improve their communicative competences with patients in another study using a mandatory course [15]. Furthermore, it is noteworthy, that in our study the use of plain language was rated to be significantly more frequent in the WHI group by a blinded expert, by the SPs, and by self-assessment of the participants while in prior research self-assessment and expert ratings of communication skills were shown to differ [32].

While the blinded expert rated the WHI group significantly better with respect to the use of stylistic devices, there was no difference with respect to comprehensibility between the two groups. The focus of the website https://washabich.de is explicitly only on “translating” medical documents [20]. Furthermore, peer supervisors train new students on the platform in the use of stylistic devices for plain language, which might explain why WHI students used them more frequently in the simulated physician-patient encounters. It might also be a sign of continuous exercise and feedback as has been shown for workplace based assessment [33]. Students do not explicitly learn to explain possible treatment options during their training at https://washabich.de. This might explain why there is no difference between the two groups with respect to comprehensibility.

In contrast to our original hypothesis that WHI participants might have gathered more medical knowledge by translating medical documents, we found no difference in medical correctness between the two groups for all medical encounters. These results resemble another study, which found no correlation between empathy and history-taking skills in simulated physician-patient encounters [34]. Both studies show, that different constructs (i.e. medical knowledge and communication skills or empathy and history-taking skills) have to be learned in different ways and integrated in a separate step for clinical practice. The great difference between the six different topics of the encounters in our current study with respect to medical correctness in the counselling might be due to the content alignment of the assessment with the prominence of the topics as learning objectives for undergraduate medical training [35].

Plain language is a prerequisite for shared decision making competences like “listing the different options” or “explaining the pros and cons of options” for a treatment decision [36]. The SPs in our study would choose a WHI participant significantly more frequently as their “personal physician” although there was no difference in satisfaction with medical counselling between both groups. This underscores the finding that SPs’ ratings in OSCEs are socially constructed and hence make absolute objectivity or standardisation impossible [24]. However, the item “I would choose this participant as my personal physician” correlated with the SPs’ ratings in the three different categories of communication skills in our study. This suggests that aspects of communication – presumably not consciously known to the SP – might play a role in choosing a participant as personal physician. If this hypothesis was tested in another study to be correct it would have great implications on supporting exercises for medical students to use plain language. Furthermore, the students’ intrinsic motivation to participate in the volunteer work on the website https://washabich.de might have contributed to their success in the assessment [37].

A strength of our study is the assessment of communication skills from three different perspectives, the SPs’ , the experts’ , and the students’. The newly developed OSCE-like assessment format for communication skills allowed to include participants independent of their residence at the time of the assessment, resembling a telecommunication exercise. Furthermore, participants were controlled for demographic variables (semester, gender) and randomized throughout the assessment and scoring with respect to being participants from the WHI or non-WHI group which strengthened the internal validity. However, pre-test communication skills were not tested. Hence, there might have been a bias towards the WHI group with a greater interest in communication and better communications skills which threatens the external validity. In addition, the study outline with its quasi-experimental design bears a threat to external validity in itself. Even though our questionnaires were based on questionnaires and scoring systems from other studies [27–29], it is a weakness of our study that our questionnaires and scoring sheets were not validated. As students participated voluntarily in this study on a “first come, first served” basis there might be a selection bias in only students with a particular interest in communication having been attracted to participate. However, since students from the WHI group have already shown a particular interest in communication by working as volunteers on the website https://washabich.de a control group with participants with a particular interest in a communication assessment might compensate for this bias.

## Conclusion

Using plain language is a prerequisite for successful physician-patient communication and shared decision making. Voluntary translation of medical documents is associated with a significantly more frequent use of plain language in simulated physician-patient encounters. There is no correlation between the use of plain language with medical correctness of the counselling. However, simulated patients would choose medical students with additional training in written communication skills more frequently as their personal physician. Further research is needed to investigate whether extracurricular written exercises can be a useful supplement for undergraduate medical students to enhance their communication skills with respect to using plain language in physician-patient communication.

## Additional file

Additional file 1:
**Original questionnaires.** (DOCX 13 kb)
