# Supplementary material for: Translating medical documents improves students’ communication skills in simulated physician-patient encounters
Source: BMC Med Educ. 2016 Feb 27;16:72. doi: 10.1186/s12909-016-0594-4 (PMC4769511; doi:10.1186/s12909-016-0594-4)
Supplement: Additional file 1: — Original questionnaires. (DOCX 13 kb) [file 12909_2016_594_MOESM1_ESM.docx]

| **Patient’s Questionnaire** | | I strongly agree | I strongly disagree |
| --- | --- | --- | --- |
| 1 | The participant used plain language. | 0 0 0 0 0 | |
| 2 | The participant explained medical terms. | 0 0 0 0 0 | |
| 3 | The participant explained the meaning of the medical report. | 0 0 0 0 0 | |
| 4 | The participant asked me whether I had understood the explanations. | 0 0 0 0 0 | |
| 5 | The participant encouraged me to ask questions. | 0 0 0 0 0 | |
| 6 | The participant answered my questions satisfactory. | 0 0 0 0 0 | |
| 7 | The participant comprehensibly explained the next steps of diagnostic or therapeutic procedures. | 0 0 0 0 0 | |
| 8 | I am satisfied with the medical counselling. | 0 0 0 0 0 | |
| 9 | I would choose this participant as my personal physician. | 0 0 0 0 0 | |

| **Participant’s Questionnaire** | | I strongly agree | I strongly disagree |
| --- | --- | --- | --- |
| 1 | I used plain language. | 0 0 0 0 0 | |
| 2 | I explained the medical terms. | 0 0 0 0 0 | |
| 3 | I explained the meaning of the medical report. | 0 0 0 0 0 | |
| 4 | I asked the patients whether they had understood the explanations. | 0 0 0 0 0 | |
| 5 | I encouraged the patients to ask questions. | 0 0 0 0 0 | |
| 6 | I answered the questions satisfactory. | 0 0 0 0 0 | |
| 7 | I comprehensibly explained the next steps of diagnostic or therapeutic procedures. | 0 0 0 0 0 | |
| 8 | I am satisfied with the medical counseling for my patients. | 0 0 0 0 0 | |
| 9 | I used plain language. | 0 0 0 0 0 | |

| **Expert’s Questionnaire** | | I strongly agree | I strongly disagree |
| --- | --- | --- | --- |
| 1 | The participant used plain language. | 0 0 0 0 0 | |
| 2 | The participant explained medical terms. | 0 0 0 0 0 | |
| 3 | The participant explained the meaning of the medical report. | 0 0 0 0 0 | |
| 4 | The participant asked the patient whether he had understood the explanations. | 0 0 0 0 0 | |
| 5 | The participant encouraged the patient to ask questions. | 0 0 0 0 0 | |
| 6 | The participant answered the patients’ questions satisfactory. | 0 0 0 0 0 | |
| 7 | The participant comprehensibly explained the next steps of diagnostic or therapeutic procedures. | 0 0 0 0 0 | |
